# Leaf litter chemistry and its effects on soil microorganisms in different ages of *Zanthoxylum planispinum* var. *Dintanensis*

**DOI:** 10.1186/s12870-023-04274-z

**Published:** 2023-05-18

**Authors:** Yanping Song, Yanghua Yu, Yitong Li, Mingfeng Du

**Affiliations:** School of Karst Science / State Engineering Technology Institute for Karst Decertification Control, Guizhou Normal University, Guiyang, 550001 China

**Keywords:** Leaf litter chemistry, Soil microorganisms, *Zanthoxylum planispinum* var. *Dintanensis* plantation, Redundancy analysis

## Abstract

**Background:**

Leaf litter is the products of metabolism during the growth and development of plantation, and it is also an important component of nutrient cycling in plantation ecosystems. However, leaf litter chemistry and its effects on soil microorganisms in different ages, as well as the interactions between chemical components in leaf litter have been rarely reported. Based on this, this paper took *Zanthoxylum planispinum* var. *dintanensis* (hereafter *Z*. *planispinum*) plantations of 5–7, 10–12, 20–22, and 28–32 years old as the objects. By using one-way ANOVA, Pearson correlation analysis and redundancy analysis, we investigated leaf litter chemistry and its effects on soil microorganisms in different ages, and to reveal internal correlation of various chemical components in leaf litter, which can provide a scientific basis for the regulation of soil microbial activity in plantations.

**Results:**

The variation of organic carbon with plantation age was more stable compared to total nitrogen and phosphorus of leaf litter. Nitrogen resorption was stronger than phosphorus resorption efficiency in *Z*. *planispinum*, and resorption efficiencies of leaf nitrogen and phosphorus for different ages were lower than the global average. Total nitrogen was highly significantly positively correlated with lignin, and total potassium was significantly positively correlated with tannin, suggesting the increase of inorganic substances in leaf litter would promote the accumulation of secondary metabolites. The leaf litter chemical traits explained up to 72% of soil microorganisms, where lignin was positively correlated with fungi and negatively correlated with bacteria, indicating that fungi are able to decompose lower quality litter and can break down complex and stable organic compounds more rapidly than bacteria. The nutrient elements carbon and nitrogen in the leaf litter and their interrelationship also have a great impact on soil microorganisms, because carbon is not only the element that provides energy, but also the element with the largest content in the microbiota.

**Conclusions:**

The sustained increase in inorganic nutrients of leaf litter did not favor the decomposition of secondary metabolites, but rather inhibited the degradation of leaf litter. The significant positive effect of the leaf litter chemistry on soil microorganisms indicates the important role of leaf litter in promoting nutrient cycling in *Z*. *planispinum* plantations.

## Introduction

Forest litter, including fallen leaves, twigs, seeds, and other woody debris, is the link between forest and soil systems, in which the leaf litter is the main component, accounting for more than 70% of the litter [1]. Because of the rapid decomposition rate of leaf litter, nutrient elements from plants can be rapidly returned to the soil. Leaf litter can provide at least 90% of nitrogen, phosphorus, and 60% of medium and trace mineral elements, providing a source of nutrients for plant root uptake and a source and reservoir of soil fertility in forest land. Leaf litter is important for nutrient cycling and energy flow in forest ecosystems [2, 3], and plays a key role in improving nutrient availability, enhancing soil fertility, maintaining plant growth and development, accelerating soil organic matter formation, and increasing net carbon storage in forest land [4, 5].

In leaf litter–soil ecosystems, nutrient release from leaf litter is a soil-microbe-mediated decomposition process in which soil microbial communities secrete various enzymes that mineralize complex organic matter into small molecules that are easily absorbed by plants [6, 7]. At the same time, leaf litter provides abundant energy and nutrient inputs for microbial growth and reproduction, and participates in their metabolic processes, thus, influencing the abundance and diversity of soil microorganisms [8, 9]. The effects of leaf litter on soil microorganisms are inconsistent among different tree species due to the variability in composition and secondary metabolites [10]. For example, Hu et al. found through in situ experiments that the increase of leaf litter produced a nitrogen stress effect, resulting in a decrease in the nitrogen content of microbial biomass in the soil [11]. Pei et al. [12] found that the number of soil fungi and actinomycetes increases with the increase of leaf litter types in a leaf litter decomposition experiment in subtropical southeast China, due to the combined chemical characteristics that likely provided more resources and microhabitat heterogeneity conditions for decomposers. Sun et al. added different types of leaf litter to soil planted with *Panax ginseng* and found that because broadleaf forests contained richer carbon source species they had higher soil microbial diversity than coniferous forests [13]. Kowallik et al. showed that yeasts were more abundant in oak leaf litter than oak bark, and that leaf litter provided a stable habitat for them because it was rich in lignocellulosic complex polysaccharides, which are broken down by fungi and bacteria into monosaccharides that can be used directly by yeasts [14]. Previous research results show that different leaf litter has significant effects on soil microbial communities, which not only affect the diversity of microbial communities, but also influence the relative abundance of specific taxa involved in the decomposition process, thus affecting its decomposition. In recent years, much attention has been paid to the regulation mechanism of subsurface ecological processes by leaf litter. However, the above studies did not examine the ecological effects of forest age at longer time scales. Studying the ecological effects of forest age at longer time scales can reveal the leaf litter chemistry and its effects on soil microorganisms in different ages. Soil nutrient supply capacity and the nutrient absorption characteristics change with growth and development of a plantation, resulting in significant changes in leaf litter chemistry [15], which will have an impact on soil microorganisms. Therefore, the mechanism of the effect of leaf litter on soil microbial activity at different ages is worth studying.

*Zanthoxylum planispinum* var. *dintanensis* (hereafter *Z. planispinum*) is in the family Rutaceae [16]; it is a semi-deciduous shrub or small tree, 2–4.5 m high. The stems and branches have sharp, reddish-brown thorns, and the base of the thorns is wide and flat. Branchlets, shoots, and leaves are glabrous or occasionally pilose. Leaves are pinnately compound. Lobules are usually lanceolate or lanceolate elliptic, opposite or alternate, 4–9 cm long, 1.5–2.5 cm wide, smooth, and glabrous. Cymose panicles are axillary or concurrent at the top of lateral branches, with a length of 2–7 cm. The ripe pericarp is mostly olive green, with a number of conspicuously raised round punctured oil glands [17]. The study site is a typical karst plateau canyon landscape, carbonate rocks are widely distributed, nutrients are easily lost with water and soil, resulting in the soil tending to be infertile and ecologically fragile, while *Z. planispinum* is highly adaptable and easy to cultivate, and has become an important pioneer species in the study site. Since 1992, large-scale *Z*. *planispinum* planting has been promoted in the study area. Since 2002, planting has increased, and the area now exceeds 100 km^2^, forming a distinctive ecological and economic industry. *Zanthoxylum planispinum* is calcium-loving, drought-resistant, and has good soil and water conservation effects [18], and has played a significant role in the ecological restoration of stone desertification areas with a 94% erosion control rate and a 92% land desertification control rate. It has typical characteristics of “strong fragrance, pure hemp flavor and excellent quality,” with an output value of 100,000–150,000 yuan per hectare and can employ 6–10 laborers, and has won such honors as China Geographical Indication Product Protection, China Forest Food Model Brand and Guizhou Famous Brand Product. In recent years, scholars have conducted many studies on the soil properties, plant functional traits, and the relationship between the two, using *Z*. *planispinum* plantations. One study showed that *Z*. *planispinum* planting improved soil nitrogen, phosphorus, and potassium, and increased the content of soil nutrients and active components [19], while another study showed that the soil carbon, nitrogen, and phosphorus contents for *Z*. *planispinum* did not change significantly with age [20]. With the increase of age, the mineralization of soil organic carbon in a *Z*. *planispinum* plantation increased and its stability decreased [21]; and a 4-year-old plantation showed stronger soil carbon fixation capacity than a 35-year-old plantation. For plant functional traits, Rong et al. classified *Z*. *planispinum* into dry and sunny leaf types based on leaf adaptation traits [22]; by comparing the amino acid accumulation characteristics of peel from spring, summer, and fall branches; Wang et al. found that summer branches were favorable for improving the amino acid quality of peel from *Z*. *planispinum* [23]. Concerning the relationship between plant traits and soil properties, Deng et al. concluded that soil nutrient element deficiency would have inhibitory effects on *Z*. *planispinum* growth and development, for example, nitrogen deficiency would reduce chlorophyll synthesis and decrease the net leaf photosynthetic rate, and phosphorus and potassium deficiency would reduce leaf stomatal conductance and inhibit the net leaf photosynthetic rate and transpiration rate [24]. Li et al. found that soil moisture had the greatest effect on leaf functional traits, such as specific leaf area and stable carbon isotope abundance, in *Z*. *planispinum*, followed by nitrogen, potassium, and calcium contents, through relative importance and redundancy analysis [25].

The above studies provide a scientific basis for management of *Z*. *planispinum*. Although the annual leaf litter amount of *Z*. *planispinum* plantations is relatively small and is rich in cellulose making it difficult to decompose, they have a strong chemosensory effect due to their rich variety of secondary metabolites [26]. Thus, it is necessary to systematically study the changes in leaf litter chemistry and the effects on soil microorganisms during growth and development of *Z*. *planispinum plantations*. This paper investigates *Z*. *planispinum* plantations of different ages. We use *Z*. *planispinum* at different ages as the study object, and propose the following hypotheses: (1) the leaf litter carbon, nitrogen and phosphorus and the nitrogen and phosphorus resorption efficiency will show a significant change with the age in the *Z*. *planispinum*, and (3) leaf litter chemistry has significant positive effects on soil microorganisms.

## Materials and methods

### Overview of the study area

The study area is in Bashan Village, Huajiang Town, Guanling County, Anshun City, Guizhou Province, China (central coordinates of 105°40′28.33″E, 25°37′57.41″N), with an elevation of 621 m. It has a subtropical humid monsoon climate, with annual rainfall of about 1100 mm unevenly distributed in seasons (severe drought in winter and spring); annual average temperature is 18.4 °C. It has valley terrain, with large height differences of about 943 m and groundwater depth of 50–100 m. Rocky desertification is developed, and the exposed rate of bedrock is more than 70%, with most of the area having a moderate and severe rocky desertification level. The area is dominated by limestone-formed soil, rich in calcium, magnesium, and other elements, with a shallow soil layer and discontinuous soil cover. This results in weak soil water holding capacity due to the development of aboveground and underground dual structure system.

### Sample plot setting

In July 2020, through detailed field survey and consulting the local forestry bureau, we selected 5–7-, 10–12-, 20–22-, and 28–32-year-old *Z*. *planispinum* plantations as study plots with similar site conditions, representing the stages of young, middle-age, mature, and over-mature plantations, respectively. Due to the fragmentation and high heterogeneity of habitats in these karst areas, three 10 m × 10 m sample plots were set up as three replications for the different ages, the corners of the sample plots were fixed with iron sticks and wooden stakes, making a total of 12 sample plots with a buffer zone of more than 5 m between each plot. The slope positions of the plantations were middle and lower sunny slopes, with slopes of 5–10°. The soil type was limestone soil. In each plot, the organic fertilizer, consisting mainly of pig manure, cow manure, grass ashes and plant leaves, was applied once a year between November and December as a base fertilizer at a dosage of about 2 kg/plant. A compound fertilizer, whose main component is nitrogen, phosphorus and potassium (15:15:15), is applied once a year from January to February, April to May, August and November, each time at a dosage of 350—450 g/plant. Because replanting can occur in the process of stand cultivation, the plantation ages are interval values rather than specific values (Table 1).

**Table 1:** Basic characteristics of sample plots

Plantation age (years)	Tree height (m)	Density (plants/hm^2^)	Crown (m)	Canopy density	Annual amount of leaf litter (kg/plant)
5–7	3.0	1150	3 × 3	0.75	1
10–12	3.0	1150	3 × 3	0.75	1
20–22	3.5	1000	3.5 × 3.5	0.55	0.8
28–32	4.2	650	4 × 5	0.5	0.6

### Sample collection and processing

#### Leaf collection and determination

In late December 2020, 3–5 plants with good growth, consistent height and crown width were randomly selected from each sample plot. Leaf litter included naturally fallen leaves and yellow leaves; in each sample plot, naturally fallen leaves (newly natural fallen undecomposed leaf litter) were collected under the *Z*. *planispinum* canopy, and yellow leaves that had not yet fallen were collected in the middle of the canopy (the standard was that they fell off with a light touch). A total of 12 samples were obtained by mixing the leaf litter collected from each sample plot, each of which was about 200 g. Leaf litter was dried in an oven for 10 min at 105 °C, then at 60 °C to a constant weight, and then crushed and sieved at 0.25 mm for storage. Mature leaves were collected in mid-June of the same year to calculate the resorption rate of nitrogen and phosphorus – these data came from Song et al. [27], so the results were not analyzed here (Table 2).

**Table 2:** Nitrogen and phosphorus content of mature leaves of *Z. planispinum* of different ages

Indicator	5–7	10–12	20–22	28–32
Leaf nitrogen (g/kg)	23.70 ± 0.57a	22.25 ± 0.50a	21.00 ± 1.98a	22.10 ± 2.97a
Leaf phosphorus (g/kg)	1.64 ± 0.35a	1.02 ± 0.23a	1.67 ± 0.40a	1.63 ± 0.25a

Note: Sample size is three. Data are presented as mean ± standard deviation. Different letters indicate significant differences among plantation ages at *P* < 0.05

The decomposition rate of leaf litter was not determined in this study, because the study area is in a dry and hot environment, and the leaves are thick leathery and rich in cellulose, with a slow decomposition rate. The organic carbon in leaf litter was determined by potassium dichromate oxidation external heating method, Total nitrogen was determined by semi-micro Kjeldahl nitrogen method after sulfuric acid–perchloric acid digestion, Total phosphorus was determined by sulfuric acid–perchloric acid digestion molybdenum–antimony antiluminosity-ultraviolet spectrophotometry, Total potassium was determined by flame spectrophotometry [28]. Soluble sugars, starch, hemicellulose, cellulose, and lignin were determined by the method of Van Soest [29] using a fiber analyzer (A2000I, ANKOM, New York, USA). The total phenol content was determined by Folin–Ciocalteu colorimetric method [30]. The tannin content was determined using vanillin hydrochloric acid method.

#### Soil microorganism collection and determination

Soil bacterial, fungal, and actinomycete quantities, and soil microbial biomass carbon, nitrogen, and phosphorus were collected and measured from Yu et al. [31], and the data are shown in Table 3.

**Table 3:** Soil microbial quantity and biomass

	Plantation age (years)			
Indicator	5–7	10–12	20–22	28–32
Bacteria (×10^5^ CFU/g)	7.90 ± 2.82a	4.55 ± 2.05a	4.95 ± 2.40a	3.30 ± 1.41a
Fungi (×10^3^ CFU/g)	3.10 ± 1.52a	2.10 ± 0.42a	4.20 ± 0.57a	4.15 ± 0.50a
Actinomycete (×10^5^ CFU/g)	4.45 ± 1.63a	2.75 ± 0.21a	2.85 ± 1.20a	2.20 ± 0.282a
Microbial biomass carbon (mg/kg)	386.00 ± 1.41a	363.00 ± 49.50a	337.50 ± 16.26a	322.50 ± 4.95a
Microbial biomass nitrogen (mg/kg)	13.05 ± 0.21c	16.70 ± 2.40b	26.15 ± 0.35a	18.95 ± 0.50b
Microbial biomass phosphorus (mg/kg)	260.00 ± 125.15ab	359.50 ± 135.06a	314.50 ± 53.03ab	52.60 ± 20.93b

Note: Sample size is three. Data are presented as mean ± standard deviation. Different letters indicate significant differences among plantation ages at *P* < 0.05

### Data processing

The formula for nutrient resorption of each element follows [32]:

$$\begin{array}{c}Nutrient\,resorption\,efficiency\,\left( \% \right){\mkern 1mu} \\= {\mkern 1mu} \left( {{W_1}{\mkern 1mu} - {\mkern 1mu} {W_2}} \right){\mkern 1mu} /{\mkern 1mu} {W_1}{\mkern 1mu} \times {\mkern 1mu} 100{\mkern 1mu} \% ,\end{array}$$

where W_1_ is the nutrient concentration of mature leaves and W_2_ is the nutrient concentration of leaf litter.

Data were first processed using Microsoft Excel (version 2013, Microsoft, Redmond, WA, USA) and statistically analyzed using SPSS (version 20.0, IBM SPSS, Armonk, NY, USA). The Kolmogorov–Smirnov method was used to test the normal distribution of the data. For data that were normally distributed, one-way ANOVA and least significant difference were applied. For non-normally distributed data, Dunett’s T3 method was used. Pearson correlation analysis was applied to the interrelationships among leaf litter chemical traits. The leaf litter chemical traits that had a strong influence on soil microorganisms were selected using principal component analysis, and then Canoco 4.5 (version 4.5, Microcomputer Power, Ithaca, NY, USA) was used for redundancy analysis to investigate the magnitude of the effect of leaf litter chemistry on soil microorganisms. Drawings were performed using Origin 8.6 (version 8.6, Originlab Corporation, Hampton, USA). Data are presented as mean ± standard deviation. Coefficient of variation of leaf litter chemistry = standard deviation/mean × 100%.

## Results

### Leaf litter chemical traits for different plantation ages

#### Characteristics of easily decomposable substances in leaf litter

**Fig. 1:**
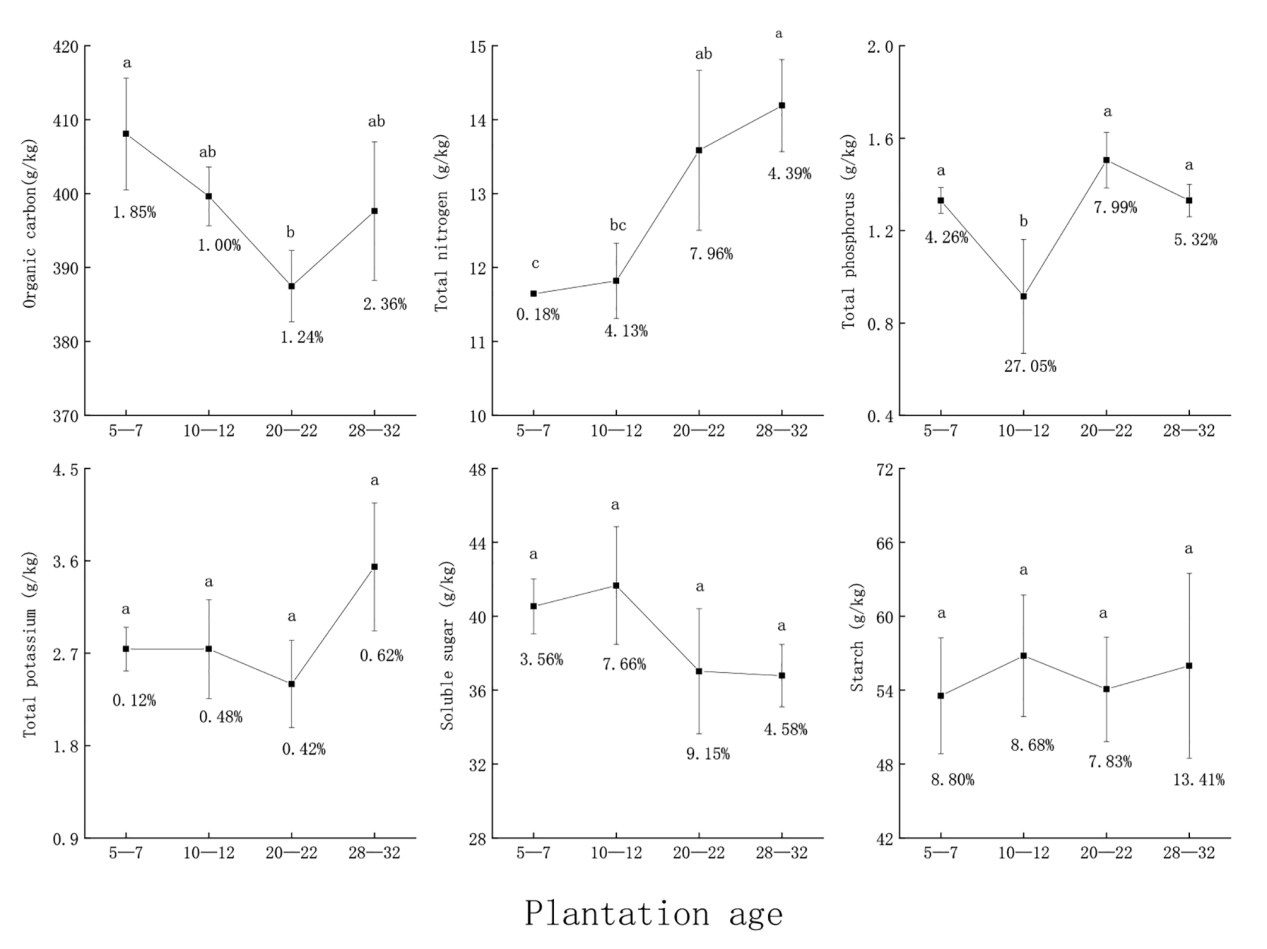
Content of easily decomposable substances in leaf litter. (Note: Sample size is three. Data are presented as mean ± standard deviation. Different letters indicate significant differences among plantation ages at *P* < 0.05. The values below the error bars are the coefficients of variation)

The organic carbon was significantly lower in the 20–22-year (387.48 ± 4.81 g/kg) than the 5–7-year group (408.72 ± 7.57 g/kg), and not significantly different from those for 10–12- and 28–32-year groups. Total nitrogen was 11.65 ± 0.02 to 14.19 ± 0.62 g/kg, significantly lower in the 5–7- than in 20–22- and 28–32-year groups, but not significantly different from the 10–12-year group. Total phosphorus was 0.92 ± 0.25 g/kg in the 10–12-year group, which was significantly lower than that of the other three groups. The total potassium, soluble sugar, and starch were 2.40 ± 0.42 to 3.54 ± 0.62, 37.03 ± 3.39 to 40.54 ± 1.48, and 54.08 ± 4.24 to 56.79 ± 4.93 g/kg, respectively, with no significant differences with plantation age. The coefficients of variation of easily decomposable substances of leaf litter in different plantation ages were 0.18–7.05%, which were mostly low. The effect of the growth and development process of *Z. planispinum* on organic carbon, total nitrogen, and total phosphorus was significant, but there was little effect on total potassium, soluble sugar, and starch (Fig. 1).

#### Characteristics of refractory substances in leaf litter

**Fig. 2:**
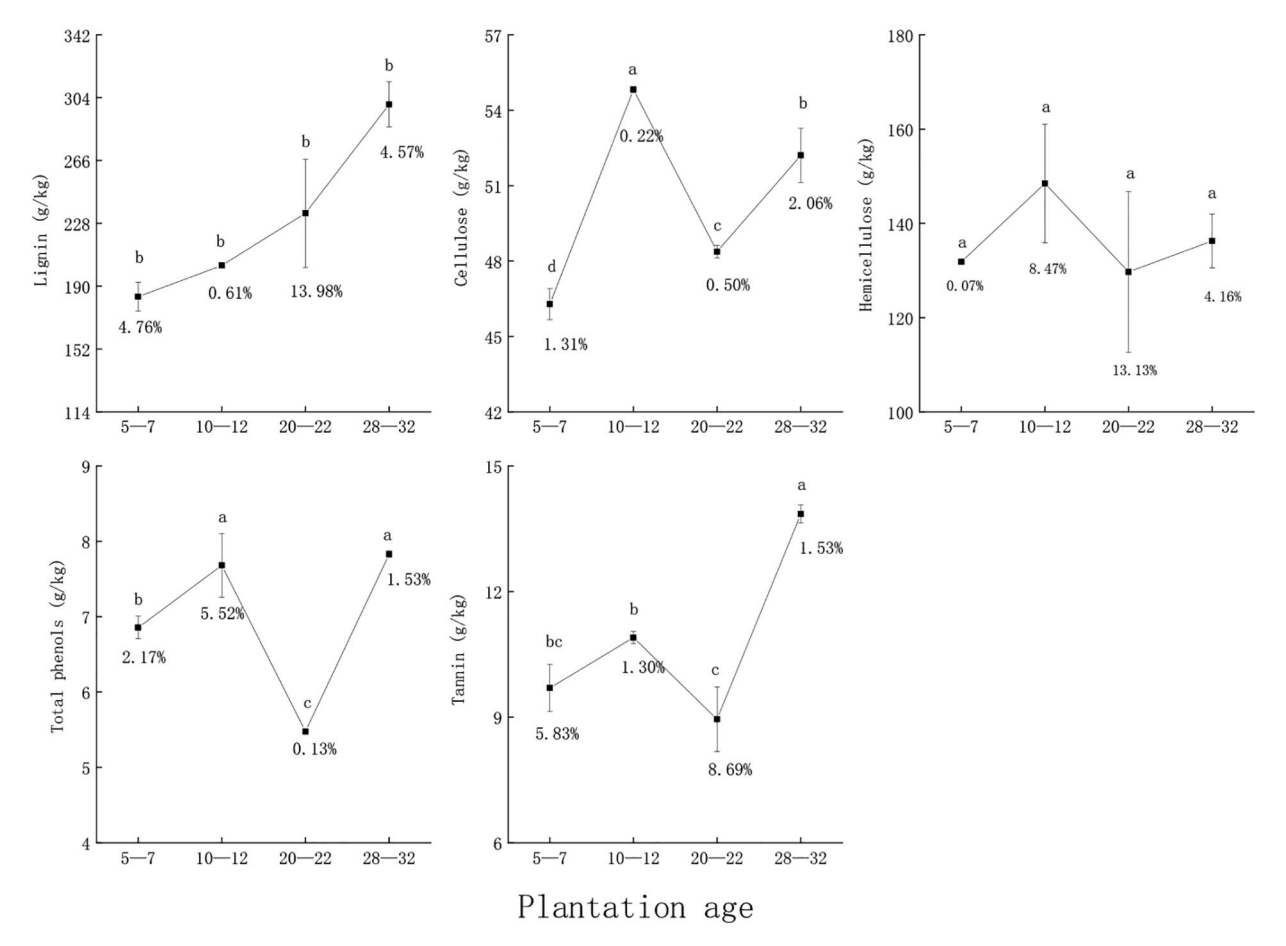
Content of refractory substances in leaf litter. (Note: Sample size is three. Data are presented as mean ± standard deviation. Different letters indicate significant differences among plantation ages at *P* < 0.05. The values below the error bars are the coefficients of variation)

Lignin and tannin were 299.92 ± 13.71 and 13.85 ± 0.21 g/kg, respectively, in the 28–32-year group, which were significantly higher than for the other three age groups. Hemicellulose was 129.66 ± 17.02 to 148.48 ± 12.57 g/kg, and did not change significantly with age. Total phenols were highest at 7.83 ± 0.04 g/kg in the 28–32-year group, which significantly differed from the 5–7- and 20–22-year groups. The highest total phenols were in the 28–32-year group (7.83 ± 0.04 g/kg), which significantly differed from the 5–7- and 20–22-year groups but not from the 10–12-year group. The coefficients of variation of refractory substances of leaf litter of different ages were 0.07–13.98%. The effect of plantation age on refractory substances in leaf litter was significant (Fig. 2).

#### Leaf litter stoichiometry characteristics

**Fig. 3:**
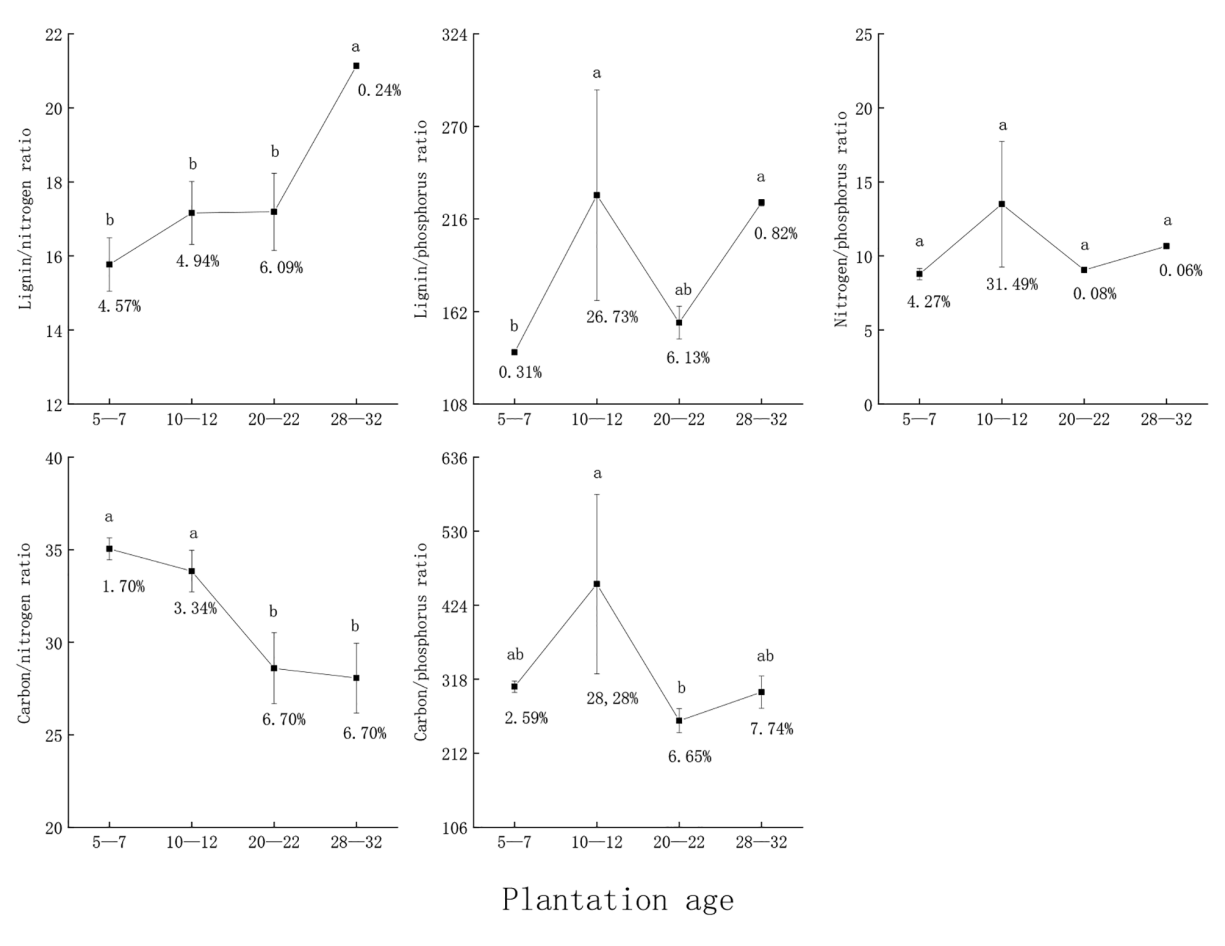
Leaf litter stoichiometry. (Note: Sample size is three. Data are presented as mean ± standard deviation. Different letters indicate significant differences among plantation ages at *P* < 0.05. The values below the error bars are the coefficients of variation)

Lignin/nitrogen ratio increased with age and was 15.77 ± 0.72 for the 28–32-year group, which was significantly higher than for the other three age groups. The lignin/phosphorus ratio was 229.76 ± 61.41 in the 10–12-year group, significantly different from the 5–7-year group but not from the other two groups. The nitrogen/phosphorus ratio was 8.78 ± 0.37 to 13.05 ± 4.25, and did not vary significantly with plantation age. The carbon/nitrogen ratio decreased with increasing plantation age, being highest in the 5–7-year group (35.04 ± 0.59), significantly different from the 20–22- and 28–32- but not from the 10–12-year group. The carbon/phosphorus ratio was significantly higher in the 10–12-year group (454.23 ± 128.45) than in the 20–22-year group and was not significantly different from the other groups. The coefficients of variation for leaf litter stoichiometry characteristics were 0.06–31.49%, which was generally low, but the 10–12-year plantation mostly exhibited large variation. The leaf litter stoichiometry characteristics changed more with increasing age (Fig. 3).

#### Characteristics of leaf nutrient transfer

**Fig. 4:**
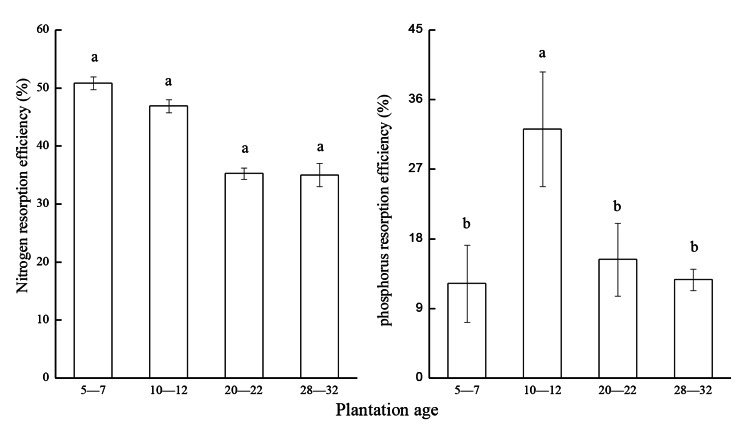
Leaf nitrogen and phosphorus resorption efficiency. (Note: Sample size is three. Data are presented as mean ± standard deviation. Different letters indicate significant differences among plantation ages at *P* < 0.05)

Since the nutrient transfer in this paper is a process of nutrient transport from leaf litter to fresh leaves, it is represented by leaf nutrient resorption. Nitrogen resorption efficiency was 35.02 ± 2.00% to 50.85 ± 1.09%, decreasing with increasing age, but with no significant changes. The phosphorus resorption efficiency was 12.24 ± 5.00% to 32.20 ± 7.40%, which is significantly higher than the other three forest age in 10—12-year group (Fig. 4).

### Trade-off and synergistic relationship among leaf litter chemistry

Lignin/phosphorus and nitrogen/phosphorus ratios were significantly positively correlated with cellulose and hemicellulose contents, and carbon/phosphorus ratio was significantly positively correlated with cellulose content, indicating that phosphorus-related traits had a greater effect on the refractory substances. Total phosphorus was highly significantly negatively correlated with carbon/phosphorus ratio; total nitrogen was positively correlated with lignin/nitrogen ratio and significantly negatively correlated with carbon/nitrogen ratio; and nitrogen/phosphorus ratio was significantly negatively correlated with total phosphorus and significantly positively correlated with lignin/phosphorus and carbon/phosphorus ratios. This showed strong relationships among nutrient stoichiometry characteristics: lignin showed a highly significant enhancement effect with total nitrogen and an inverse effect with carbon/nitrogen ratio, hemicellulose showed a significant positive correlation with soluble sugars, tannin increased with accumulation of total potassium and total phenols, and the positive correlation between lignin/nitrogen ratio and lignin and tannin reached a highly significant level. The results showed that organic carbon, total phosphorus, and starch were not related to other traits (i.e., without their own stoichiometry), and there were mostly positive correlations among leaf litter chemistry, indicating strong coordination among traits (Table 4).

**Table 4:** Correlations for leaf litter chemistry

Indicator	Organic carbon	Total nitrogen	Total phosphorus	Total potassium	Soluble sugars	Starch	Lignin	Cellulose	Hemicellulose	Total phenol	Tannins	Lignin/nitrogen ratio	Lignin/phosphorus ratio	Nitrogen/phosphorus ratio	Carbon/nitrogen ratio
Total nitrogen	−0.506														
Total phosphorus	−0.330	0.495													
Total potassium	0.481	0.392	−0.160												
Soluble sugars	0.545	−0.433	−0.571	0.007											
Starch	0.212	−0.267	−0.162	0.204	−0.341										
Lignin	−0.332	0.917**	0.340	0.589	−0.456	−0.018									
Cellulose	−0.060	0.053	−0.685	0.342	0.116	0.434	0.291								
Hemicellulose	0.226	0.042	−0.637	0.313	0.773*	−0.308	0.079	0.562							
Total phenol	0.450	−0.139	−0.544	0.546	0.210	0.314	0.236	0.651	0.412						
Tannins	0.104	0.400	−0.211	0.718*	−0.236	0.266	0.701	0.566	0.164	0.770*					
Lignin/nitrogen ratio	−0.179	0.754*	0.181	0.667	−0.430	0.207	0.952**	0.466	0.102	0.483	0.839**				
Lignin/phosphorus ratio	0.031	0.285	−0.679	0.597	0.246	0.067	0.440	0.838**	0.714*	0.586	0.664	0.515			
Nitrogen/phosphorus ratio	0.123	−0.069	−0.883**	0.325	0.525	−0.040	−0.002	0.724*	0.784*	0.407	0.305	0.055	0.884**		
Carbon/nitrogen ratio	0.661	−0.980**	−0.488	−0.249	0.513	0.255	−0.868**	−0.070	0.025	0.232	−0.327	−0.692	−0.257	0.068	
Carbon/phosphorus ratio	0.335	−0.423	−0.977**	0.182	0.664	0.055	−0.330	0.630	0.721*	0.430	0.132	−0.218	0.700	0.931**	0.424

### Regulation of leaf litter chemical traits on soil microorganisms

#### Principal component analysis of leaf litter chemistry

Principal component analysis was performed on 15 indicators (Table 5). There were four principal components with eigenvalues > 1, and their cumulative contribution reached 93.731%. The factors with loadings > 0.8 were extracted and further analyzed for their influence on soil microorganisms. Principal component 1 was dominated by total phosphorus, cellulose, hemicellulose, and lignin/phosphorus, nitrogen/phosphorus, and carbon/phosphorus ratios; principal component 2 was more influenced by total nitrogen, lignin, and lignin/nitrogen and carbon/nitrogen ratios; principal component 3 was dominated by organic carbon; and principal component 4 was influenced by starch. The principal component analysis screened out 12 major traits: organic carbon, total nitrogen, total phosphorus, starch, lignin, cellulose, hemicellulose, and lignin/nitrogen, lignin/phosphorus, nitrogen/phosphorus, carbon/nitrogen, and carbon/phosphorus ratios.`.

**Table 5:** Principal component analysis of leaf litter chemical traits

Indicator	Principal components			
	1	2	3	4
Organic carbon	0.060	−0.388	**0.903**	−0.033
Total nitrogen	−0.120	**0.945**	−0.177	−0.216
Total phosphorus	**−0.902**	0.339	−0.169	−0.139
Total potassium	0.225	0.561	0.703	0.077
Soluble sugars	0.523	−0.460	0.354	−0.560
Starch	0.021	−0.093	0.164	**0.897**
Lignin	−0.014	**0.994**	0.064	0.058
Cellulose	**0.823**	0.263	0.014	0.443
Hemicellulose	**0.808**	0.084	0.225	−0.450
Total phenol	0.488	0.174	0.636	0.381
Tannins	0.308	0.666	0.436	0.402
Lignin/nitrogen ratio	0.080	**0.920**	0.217	0.281
Lignin/phosphorus ratio	**0.869**	0.448	0.156	0.065
Nitrogen/phosphorus ratio	**0.970**	0.024	0.051	−0.088
Carbon/nitrogen ratio	0.106	**−0.906**	0.363	0.160
Carbon/phosphorus ratio	**0.920**	−0.318	0.152	−0.026
Eigenvalue	6.132	5.276	2.168	1.421
Variance contribution (%)	33.626	32.495	15.003	12.606
Total cumulative (%)	33.626	66.121	81.125	93.731

Bolded values indicate high load values

#### Effects of leaf litter chemical traits on soil microorganisms

**Fig. 5:**
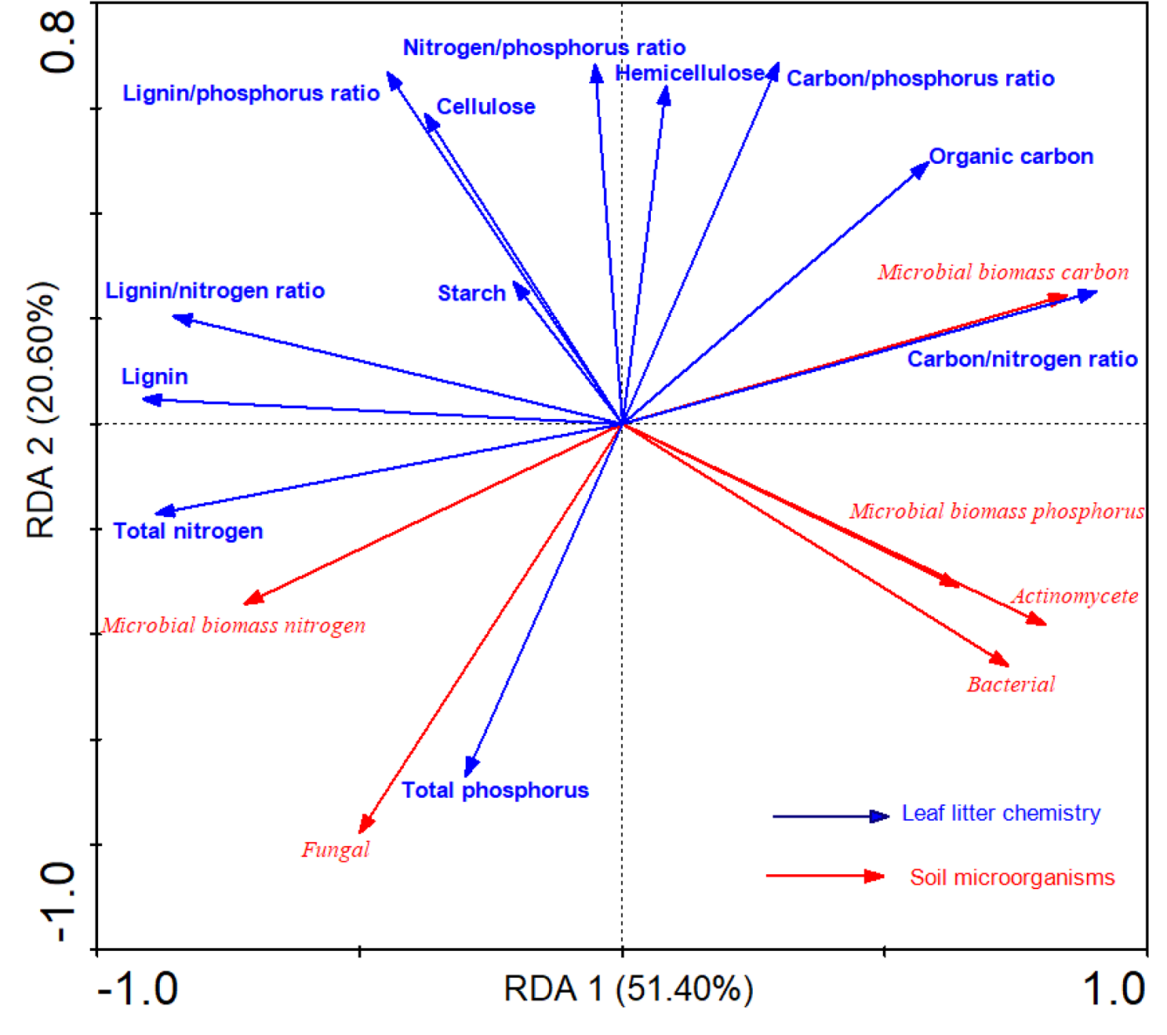
Redundancy analysis of leaf litter and soil microorganisms

Redundancy analysis was conducted with leaf chemistry as explanatory variables (blue arrows) and soil microorganisms as response variables (red arrows). The length of the lines connecting the leaf litter trait arrows represent the magnitude of the effect on soil microorganisms. The size of the angles between the lines connecting the leaf litter chemical traits and soil arrows indicate the level of correlation between them: acute angles indicate positive correlations, obtuse angles indicate negative correlations, and angles close to 90° indicate lack of correlation. leaf litter chemical traits explained a large amount (72%) of soil microorganism variation by axis I and axis II (Fig. 5; Table 6). Organic carbon, total nitrogen, carbon/nitrogen ratio, lignin, and lignin/nitrogen ratio had large and significant effects on soil microorganisms, while other leaf litter chemistry traits had little effect. Microbial biomass carbon, microbial biomass phosphorus, actinomycetes, and bacteria increased with organic carbon and carbon/nitrogen ratio; microbial biomass nitrogen and fungi increased with the accumulation of total nitrogen, total phosphorus, lignin, and lignin/nitrogen ratio; and the increase of lignin and lignin/nitrogen ratio led to the decrease of microbial biomass carbon and nitrogen, actinomycetes, bacteria. The results showed that leaf litter carbon and nitrogen and their stoichiometric relationships had the greatest effects on soil microorganisms. The effect of starch on microorganisms was small.

**Table 6:** Interpretation and significance test of leaf litter chemistry

Indicator	Explanation rate (%)	*P*
Lignin	15.01	0.002
Carbon/nitrogen ratio	14.74	0.002
Total nitrogen	14.24	0.002
Lignin/nitrogen ratio	13.77	0.002
Organic carbon	9.27	0.016

## Discussion

### Leaf litter chemistry varies with plantation age

In this study, the nitrogen and phosphorus contents in the leaf litter were higher in the late growth period than in the early growth period. According to growth rate hypothesis [33], the reason may be that in the early- and mid-growth stages, the plant has a high growth rate and thus needs to consume large amounts of nitrogen and phosphorus, while the soil cannot provide sufficient nutrients (Li et al., 2018) [34]. The increase in nutrient demand causes a decrease in the nitrogen and phosphorus content of mature leaves, which leads to a decrease in the nitrogen and phosphorus nutrients of leaf litter. In the late growth stage, the growth rate of the plantation slows, leading to lower nutrient demand, so that the nitrogen and phosphorus nutrients accumulate in leaf litter. It is also possible that late growth stands are in decline, relatively less resistant to pests and diseases, and plants have increased inputs for the synthesis of protective phenolic substances such as lignin and cellulose [35], which makes it difficult for microorganisms to use them. Therefore, the leaf litter fix nitrogen and phosphorus nutrients from the environment meet the growth and reproduction needs of microorganisms [36], leading to elevated nitrogen and phosphorus contents of leaf litter. The coefficients of variation of organic carbon, total nitrogen, and total phosphorus were 1.24–2.36%, 0.18–4.39%, and 4.26–27.05%, respectively. This indicates that as growth and development of *Z. planispinum* progresses, the content of carbon-rich structural substances is more stable and the limiting effect on plant growth and development is weakened, while nitrogen- and phosphorus-rich functional substances and storage substances are more variable [37]. The first reason for this is that carbon tends to be relatively stable during the transition from maturity to leaf litter, while nitrogen and phosphorus can be reabsorbed and utilized [38]. Second, the leaves of *Z. planispinum* usually have a large amount of carbon-containing secondary metabolites (e.g. tannins, resins, and waxes) that are chemically more stable, and nitrogen and phosphorus mostly form unstable compounds [36, 39]. Our results showed that the resorption efficiencies of leaf nitrogen and phosphorus (62.10% and 64.90%, respectively) for different ages were lower than the global average [40], probably due to differences in the study area or tree species, as well as the fact that *Z. planispinum* plantations are treated with compound fertilizers (nitrogen:phosphorus:potassium = 15:15:15), which may alleviate nitrogen and phosphorus limitation to some extent. Thus, it is inferred that chemical fertilizer application may affect nutrient resorption efficiency [41], which provides a reference for fertilization measure formulation. The resorption efficiency of nitrogen was far greater than that of phosphorus, which is inconsistent with previous research [42, 43]. Firstly, we speculate that the calcium-rich geological background and the characteristics of soil erosion and leakage in karst areas lead to a general lack of phosphorus effectiveness – due to the precipitation reactions of phosphorus with calcium, iron, aluminum, etc., it is difficult for most phosphorus to be directly absorbed and used by plants [44]; Secondly, Han et al. [45] suggested that plants have a higher element resorption rate when they are limited by it during growth. A previous study by our team showed that soil in the study area was more nitrogen-limited and the leaf litter nitrogen/phosphorus ratio was less than 14 at different ages, indicating that plants were more nitrogen-limited in their bodies [46]. Therefore, both soil and the plant body were nitrogen-limited, thus the plant spontaneously enhances nitrogen resorption.

### Trade-off and synergy between leaf litter chemistry

Single-species plantations are susceptible to pests and diseases, and plants in plantations enhance their defenses by increasing the content of carbon-rich secondary metabolites such as tannins and waxes, resulting in higher carbon contents, but not conducive to the decomposition of leaf litter [47], the results of this paper confirm this, that there is a positive correlation between organic carbon and tannin. Lignin and nitrogen were significantly positively correlated because reasonable nitrogen concentration can promote key enzyme activities and facilitate lignin accumulation [48], it is also possible that the increase in nitrogen will inhibit the growth of white rot fungi, resulting in the suppression of ammonia metabolism and hindering the rate of synthesis of lignin-degrading enzymes in leaf litter, further leading to a gradual decrease in the activity of lignin oxidase degradation and inhibition of lignin breakdown [49, 50]. The results showed a significant positive correlation between inorganic and organic substances, implying that an increase in inorganic nutrient content would have an inhibitory effect on the decomposition rate of leaf litter, resulting in an inability to release nutrients rapidly. Tannins and total phenols, as organic components of leaf litter that are difficult to decompose, were positively correlated because tannins are highly aggregated compounds in total phenols, so total phenol content accumulates with increasing tannins [51]. Total nitrogen was highly significantly negatively correlated with carbon/nitrogen ratio. We speculate that in the early- and mid-growth stages of the plant, the faster growth rate leads to a large amount of nitrogen consumption, while carbon gradually accumulates in the plant through photosynthesis, resulting in a higher carbon/phosphorus ratio [33]. Our results showed no significant correlation between organic carbon and total nitrogen, consistent with the study of Ge et al. [38], probably because carbon and nitrogen are both important constituents of organic matter and part of the nitrogen is fixed in carbon-rich structural compounds, thus limiting the close correlation between organic carbon and total nitrogen [39]. The results of the study were contrary to our hypothesis that inorganic substances in leaf litter could provide nutrients for the decomposition of secondary metabolites, and the results showed that the increase of inorganic substances in leaf litter would promote the accumulation of secondary metabolites; however, the specific mechanism needs to be verified in further studies.

### Effect of leaf litter chemistry on soil microbial quantity and biomass

Leaf litter accumulating on the soil surface is the largest source of organic matter and nutrients for the humus layer [36], and also provides essential nutrients for soil microbial reproduction. As expected from hypothesis (3), our study revealed a significant effect of leaf litter chemistry on microorganisms. The leaf litter had a large effect on soil microbial concentration and biomass, explaining 72% of the variation (Fig. 5). This indicates that nutrient effectiveness in the soil is low because when the soil is effectively inadequate, it is difficult to meet the growth and reproduction needs of microorganisms and they rely more on the nutrients in exotic leaf litter [52], consistent with the general characteristics of poor soil nutrients in karst areas. The inorganic and organic nutrients contained in leaf litter are the main source of nutrients for soil microbial colonization, so the availability of nutrients in leaf litter is a limiting factor for microbial growth, which lays the foundation for regulating the type and amount of litter to improve soil microbial activity [53]. Lignin was positively correlated with fungi and negatively correlated with bacteria. In general, fungi are able to decompose lower quality litter and can break down complex and stable organic compounds more rapidly than bacteria [54]. The likely reason is that with the decomposition of leaf litter, lignin and other macromolecular organic substances that are difficult for microorganisms to use then accumulate in large quantities, leading to a decrease in organic matter and effective nutrients, and thus a decrease in the number of bacteria; because fungi degrade lignin and other refractory substances by producing a variety of effective extracellular enzymes, the number of fungi increased [55, 56]. This shows that the substrate required by soil microorganisms has important practical value. The nutrient elements carbon and nitrogen in the leaf litter and their interrelationship also have a great impact on soil microorganisms, because carbon is not only the element that provides energy, but also the element with the largest content in the microbiota [57]. Nitrogen is an essential nutrient in the process of microbial growth and reproduction, so changes in carbon and nitrogen concentration and relative proportion have a great impact on microbial proliferation. The carbon/phosphorus ratio is widely considered as one of the best predictors of litter decomposition because it reflects the ratio of carbohydrates to proteins in litter [58]; leaf litter with low carbon/nitrogen ratio is preferentially colonized by soil microorganisms because nitrogen is an essential and limiting element for microbial metabolism, and therefore leaf litter with low carbon/nitrogen ratio decomposes more rapidly [59]. When leaf litter is nitrogen deficient (high carbon/nitrogen ratio), it is generally not easily decomposed and the microbial community needs to obtain nitrogen from external sources [58]. Tannin is a phenolic substance with wide distribution and high content in plants, but its weight was lower than that of other metabolic substances in this study (Table 5). This is because the decomposition rate of tannin is slow [60] as it is difficult for soil microorganisms to use. Thus, the decomposition of leaf litter can affect soil microbial reproduction. However, since the collected leaf litter was fresh and did not fully enter the soil nutrient cycle, and a considerable portion of soil microorganisms could not directly invade the leaf litter and participate in the decomposition process. The mechanism of the impact of leaf litter decomposition on microbial growth requires further study.

## Conclusion

In this study, the leaf litter chemistry showed small fluctuations with age, which is not consistent with our first hypothesis. Such as lower nitrogen and phosphorus nutrient concentration in the early compared to the late growth stage. However, the changes in organic carbon concentration with age were more stable compared with total nitrogen and phosphorus concentration in leaf litter, indicating a greater variation of plant nitrogen- and phosphorus-rich functional substances. In order to build a defense system, *Z*. *planispinum* will increase the input of secondary metabolites such as lignin, cellulose, and tannin. Nutrient resorption efficiency was greater for nitrogen than phosphorus, and the effect of age was stable. Our second hypothesis was that the inorganic material of leaf litter would promote the decomposition of secondary metabolites, however, we concluded that the continuous increase of inorganic nutrients would not promote the decomposition of secondary metabolites, but would reduce the decomposition rate of leaf litter, and the mechanism of its influence needs to be further investigated. The leaf litter chemistry of *Z*. *planispinum* explained up to 72% of the variation in soil microorganisms, supporting the third hypothesis and indicating that in the nutrient-poor karst region, the soil microorganisms of *Z*. *planispinum* plantations depend mainly on nutrients from leaf litter.

## Data Availability

The datasets generated and analysed during the current study are not publicly available due we need to use the data from this study further, but are available from the corresponding author on reasonable request.
